# A Role for Transcription Factor GTF2IRD2 in Executive Function in Williams-Beuren Syndrome

**DOI:** 10.1371/journal.pone.0047457

**Published:** 2012-10-31

**Authors:** Melanie A. Porter, Carol Dobson-Stone, John B. J. Kwok, Peter R. Schofield, William Beckett, May Tassabehji

**Affiliations:** 1 Psychology Department, Macquarie University, Sydney, Australia; 2 Neuroscience Research Australia, Sydney, Australia; 3 School of Medical Sciences, University of New South Wales, Sydney, Australia; 4 Genetic Medicine, University of Manchester & St Mary's Hospital, Manchester, United Kingdom; University of Medicine & Dentistry of NJ - New Jersey Medical School, United States of America

## Abstract

Executive functions are amongst the most heritable cognitive traits with twin studies indicating a strong genetic origin. However genes associated with this domain are unknown. Our research into the neurodevelopmental disorder Williams-Beuren syndrome (WBS) has identified a gene within the causative recurrent 1.5/1.6 Mb heterozygous microdeletion on chromosome 7q11.23, which may be involved in executive functioning. Comparative genome array screening of 55 WBS patients revealed a larger ∼1.8 Mb microdeletion in 18% of cases, which results in the loss of an additional gene, the transcription factor *GTF2IRD2*. The GTF gene family of transcription factors (*GTF2I, GTF2IRD1* and *GTF2IRD2*) are all highly expressed in the brain, and *GTF2I* and *GTF2IRD1* are involved in the pathogenesis of the cognitive and behavioural phenotypes associated with WBS. A multi-level analysis of cognitive, behavioural and psychological functioning in WBS patients showed that those with slightly larger deletions encompassing *GTF2IRD2* were significantly more cognitively impaired in the areas of spatial functioning, social reasoning, and cognitive flexibility (a form of executive functioning). They also displayed significantly more obsessions and externalizing behaviours, a likely manifestation of poor cognitive flexibility and executive dysfunction. We provide the first evidence for a role for *GTF2IRD2* in higher-level (executive functioning) abilities and highlight the importance of integrating detailed molecular characterisation of patients with comprehensive neuropsychological profiling to uncover additional genotype-phenotype correlations. The identification of specific genes which contribute to executive function has important neuropsychological implications in the treatment of patients with conditions like WBS, and will allow further studies into their mechanism of action.

## Introduction

Williams-Beuren Syndrome (WBS) is a neurodevelopmental disorder caused by a hemizygous multi-gene deletion on chromosome 7q11.23. Clinical features are multi-systemic and include characteristic craniofacial, cardiovascular, growth and neurological abnormalities [1]–[4]. Abnormal spatial and social abilities play a role in the striking cognitive and behavioural phenotype, which includes a global intellectual impairment, with weaknesses in spatial and visual motor skills alongside strengths in auditory, verbal and short-term memory abilities. WBS individuals are also very friendly, gregarious and empathic, with relatively good emotion recognition abilities. In contrast to their good social perceptual skills, their social reasoning capabilities, such as theory of mind are impaired [5], [6]. They display poor impulse control (behavioural disinhibition), a deficit which may underlie their apparent overfriendly personalities and tendency to approach strangers [7], [8].

Global brain abnormalities in WBS include an overall reduction in brain and cerebral volumes and abnormal cortical shape [9]–[13]. Structural and functional studies indicate impairments in dorsal stream functions and in the frontostriatal and amygdala-prefrontal circuitry [14], [15].

The prevalence of WBS is 1–2 in 20,000 [16], and most cases are sporadic [17]–[18] arising from instability at the 7q11.23 locus, which contains highly repetitive segmental duplications. These low copy repeats (LCRs) make the region prone to chromosome rearrangements through a mechanism of non-allelic homologous recombination [19]. The typical deletion, encompassing a region of about 1.5–1.6 Mb (∼26 genes), occurs in >95% of cases [19], [20]. There are rare cases with deletions in the critical region involving as few as 2 genes or up to 35 genes [21]–[25], with consequential milder or more severe phenotypes, respectively. These atypical cases have enabled genotype-phenotype correlations, highlighting the roles of *LIMK1, CYLN2/CLIP2* (which regulate dynamic aspects of the cell cytoskeleton) [26], [27], and the *GTF2I* gene family as candidates for the neurological features. The *GTF2I* family, clustered at the WBS locus, encode ubiquitously expressed transcription factors with roles in many developmental pathways, making them strong candidates for the main neurological phenotypes [28]–[30]. GTF2I/TFII-I is a general transcription factor, GTF2IRD1 a determinant of craniofacial and neurological development, while the role of GTF2IRD2 is as yet undefined.

Extensive phenotypic heterogeneity has been reported in WBS patients across a range of clinical, psychological and cognitive functions [23], [31]–[38]. This heterogeneity may be a consequence of variable penetrance, differences in the genomic region deleted or additional genetic mutations occurring elsewhere in the genome. Previous studies have compared rare patients with smaller deletions to ‘classical’ cases [21]–[25], but this study is the first to compare the genotype and detailed neuropsychological capabilities of patients grouped in the ‘classical’ WBS range. Our hypothesis is that patients clinically classified as typical WBS but who harbour slightly larger ∼1.8 Mb deletions, will display additional neuropsychological features due to haploinsufficiency for additional genes,, thus explaining some of the phenotypic heterogeneity seen in WBS.

## Materials and Methods

### Genetic Analysis

Australian ‘classical WBS’ patients (n = 55) were ascertained by experienced clinical geneticists. Patients were screened for a deletion using the *ELN/LIMK1* FISH probe [39], and all had a normal karyotype apart from the 7q11.23 microdeletion. Blood for DNA analysis was obtained from patients and their parents. Ethical consent for this study was obtained from Macquarie University.

Quantitative PCR (qPCR) analysis was used to determine deletion breakpoints by using 60 probes that cover and extend into the WBS flanking LCR regions (Methods S1). Array comparative genomic hybridization (aCGH) with the Agilent International Standard Cytogenomic Array Consortium custom 8×80 k array were used to detect sub-microscopic abnormalities, copy number variants (CNVs) [40] according to manufacturer's instructions. All CNVs detected were screened against the Database of Genomic Variants (DGV) to establish whether they were polymorphisms or unique variants. Patients were grouped accoording to their deletion size: Group 1 = 1.5/1.6 Mb deletion; Group 2 = 1.8 Mb deletion.

### Neuropsychological Testing

Intellectual and more specific cognitive abilities were assessed using the Woodcock-Johnson Test of Cognitive Ability – Revised (WJ-R COG) [41], [35]. Spatial measures included a task of spatial construction [36] and spatial perception (Test 19 Spatial Relations from the WJ-R COG, see Figure S1). Social tasks included: 1) an emotion recognition task - The Diagnostic Analysis of Nonverbal Accuracy [42], [7]; 2) a social reasoning or ‘Theory of Mind’ measure, namely, a nonverbal picture sequencing task (Figure S2) [6]. Behavioural inhibition and cognitive flexibility were assessed using The Shape School Test [43], [7] (Methods S1).

General history was collected from primary caregivers using: a generic background history questionnaire; a semi-structured DSM-IV diagnostic interview that assesses 32 DSM-IV Axis I psychiatric diagnoses known as The Schedule for Affective Disorders and Schizophrenia (K-SADS-PL) [44], [45] and the Vineland Adaptive Behavior Scales – 2^nd^ Edition (Parent Survey) or Vineland-II [46]. Additional standardized measures administered to atypical patients, included the Benton Face Recognition Test [47], The Benton Judgment of Line Orientation Test [48] and the Differential Ability Scales (DAS) [49]. Where standardized measures were administered, we used the normative data available (Woodcock-Johnson Test of Cognitive Ability – Revised; K-SADS-PL; Vineland-II, Benton Face Recognition; Benton Judgment of Line Orientation; DAS). In all other instances, we included a typically developing (TD) control group matched to WBS patients on chronological age (CA) or mental age (MA).

WBS patients with a ∼1.5/1.6 Mb deletion (Group 1) were selected to individually match the ∼1.8 Mb deleted individuals (Group 2) (n = 9 in each group) on CA, IQ, Socio-Economic Status (SES), sex and hand dominance. SES was obtained using the Index of Relative Socio-Economic Advantage and Disadvantage and was representative of the Australian population (national mean±SD = 1000±100). TD controls individually matched typical WBS patients on sex and either MA or CA (Table S1). Different control participants were used for each of the non-standardised neuropsychological measures. There were no statistical differences between WBS patients and control groups on the group matching variables. We recruited separate MA- and CA-matched TD controls for atypical patients WBS425 and WBS023I, as MA and CA were higher than the typical WBS groups. Means ± SD for this new control group were not significantly different to atypical patients. No participant had a history of neurological problems not associated with WBS, depression or any other psychological illness that would affect their results.

### Statistical Analyses

Analyses of variance and follow-up one-tailed t-tests were used to explore group differences between the two WBS patient groups and between patient groups and controls. For follow-up comparison t-tests, we adjusted our alpha level to 0.01 to control for multiple comparisons. Crawford's between-groups test of single case comparison was used for statistical analyses comparing the two atypical cases to their MA- and CA-matched control group, as this procedure allows for the comparison between a single patient and their controls (n = 5) [50].

## Results

### Genetic Characterisation of Patients

**Figure 1:**
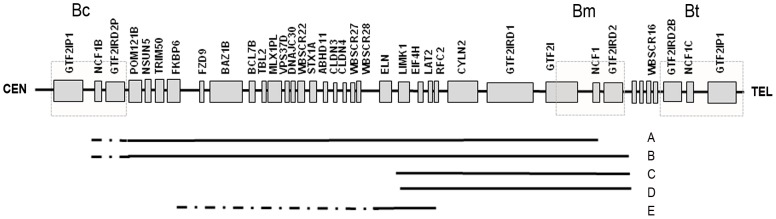
Williams-Beuren syndrome region on chromosome 7q11.23. Black lines represent the regions deleted in WBS patients. A: common ∼1.5/1.6 Mb deletion; B: ∼1.8 Mb deletion; C: patient WBS245; D: patient WBS023I; E: partial deletion patients with only isolated SVAS and associated cardiac pathologies[22], [23]. Shaded boxes represent the B-block flanking duplicons containing the deletion breakpoints (Bc = centromeric; Bm = medial; Bt = telomeric). Not to scale.

53 of the 55 WBS patients were positive for a deletion using the *ELN/LIMK1* FISH test [39]. *ELN/LIMK1* qPCR analysis on parental DNA confirmed a *de novo* mutation status in all cases. Breakpoint mapping by qPCR into the LCR flanking regions identified ten patients (∼18%) with a larger ∼1.8 Mb heterozygous deletion including *NCF1* and *GTF2IRD2* (Group 2) (Figure 1; Table S2). 43 patients (Group 1) had the 1.5/1.6 Mb heterozygous deletion. This frequency is higher than the 5% reported in the literature [20], [21], [51], but consistent with more recent estimates from our UK cohort (MT unpublished data). The two FISH-negative WBS patients (WBS023I and WBS425) harboured smaller ∼0.78 Mb deletions extending from *LIMK1* to distal breakpoints within the *GTF2IRD2* gene (Figure 1; Table 1).

**Table 1:** Clinical and genetic profile of WBS patient cohorts.

Patient ID	FISH	#Size of deletion	WBSCR	Distal gene	FD	C	SS	HCa	HA	HS	Shy	Anx	Fear	DD	IQ
	test	∼Mb	deletion	deleted											
**Controls average**	−ve	none	none	none	n	n	n	n	n	n	n	n	n	n	average
**WBS average**	+ve	1.5/1.6	Bc-Bm	*GTF2I/NCF1*	y	y	y	y	y	y	y	y	y	y	mild to moderate impairment
004B	+ve	1.5	Bc-Bm	*GTF2I*	y	y	n	n	y	y	n	y	y	y	mild to moderate impairment
030J	+ve	1.54	Bc-Bm	*NCF1*	y	y	y	n	y	y	n	y	y	y	mild impairment
072X	+ve	1.54	Bc-Bm	*NCF1*	y	n	y	n	n	y	n	y	y	y	mild to moderate impairment
081AA	+ve	1.54	Bc-Bm	*NCF1*	y	y	y	y	y	y	n	y	y	y	mild to moderate impairment
022H	+ve	1.62	Bc-Bm	*GTF2I*	y (mild)	y	y	n	y	y	n	y	y	y	borderline impairment
046P	+ve	1.62	Bc-Bm	*GTF2I*	y	y	y	n	y	y	n	y	y	y	mild impairment
051Q	+ve	1.62	Bc-Bm	*GTF2I*	y	y	n	y	y	y	n	y	y	y	mild to moderate impairment
066V	+ve	1.62	NOL1R-Bm	*GTF2I*	y	y	n	n	y	y	n	y	y	y	mild to moderate impairment
019G	+ve	1.64	Bc-Bm	*NCF1*	y	n	y	n	y	y	n	y	y	y	mild to moderate impairment
178III	+ve	1.64	Bc-Bm	*NCF1*	y	y	y	n	y	y	n	y	y	y	mild to moderate impairment
012E	+ve	1.8	Bc-Bm	*GTF2IRD2*	y	y	y	y	y	y	n	y	y	y	mild to moderate impairment
032K	+ve	1.8	Bc-Bm	*GTF2IRD2*	y	y	y	y	y	y	n	y	y	y	mild to moderate impairment
043O	+ve	1.8	Bc-Bm	*GTF2IRD2*	y	y	y	y	y	y	n	y	y	y	mild to moderate impairment
054R	+ve	1.8	Bc-Bm	*GTF2IRD2*	y	y	y	y	y	y	n	y	y	y	mild to moderate impairment
056S	+ve	1.8	Bc-Bm	*GTF2IRD2*	y	y	y	y	n	y	n	y	y	y	moderate impairment
062U	+ve	1.8	Bc-Bm	*GTF2IRD2*	y	n	y	n	y	y	n	y	y	y	mild to moderate impairment
069W	+ve	1.8	Bc-Bm	*GTF2IRD2*	y	y	y	n	y	y	n	y	y	y	severe impairment
196OOOb	+ve	1.8	Bc-Bm	*GTF2IRD2*	y	y	y	n	y	y	n	y	y	y	mild to moderate impairment
112LL	+ve	∼2	Bc-Bt	*GTF2IRD2B*	y	y	y	y	y	y	n	y	y	y	borderline to mild impairment
187LLL	+ve	∼3.3	*CALN1*-Bt	*GTF2IRD2B*	y	y	y	y	y	y	n	y	y	y	mild to moderate impairment
WBS023I	−ve	∼0.78	LIMK1-Bm	*GTF2IRD2*	y	y	n	y	y	y	n	y	y	y	borderline impairment
WBS425	−ve	∼0.78	LIMK1-Bm	*GTF2IRD2*	y	y	n	y	y	y	n	y	y	y	borderline to mild impairment

***Key:*** +ve = deleted for ELN/LIMK1 FISH probe; −ve = not deleted for ELN/LIMK1 FISH probe. Bc refers to the centromeric breakpoint and Bm to the medial breakpoint.

# approximate size due to the breakpoints residing in LCR duplicon regions. FD = facial dysmorphology; C = cardiac abnormality; SS = short stature; Hca = hypercalcaemia; HA = Hyperacusis; HS = hypersociability; Anx = Anxiety; Fear = Fears or Phobias; DD = developmental delay.

CNVs are associated with many neurological syndromes, therefore all patients were screened by genome-wide aCGH (Table S2). No additional novel or known disease-associated CNVs were detected in the patients. No *GTF2IRD2* gene deletions were identified on screening 200 control chromosomes by qPCR, indicating it is not a common CNV.

### GTF2IRD2 expression

*GTF2IRD2* is ubiquitously expressed with high expression in human foetal and adult brain (Figure S3A). *In silico* expression profiling in normal adult brain using the GNF, GEO and the Allen Brain Atlas databases [52]–[54] revealed *GTF2IRD2* expression in all main brain regions, especially cerebellum, orbitofrontal cortex and dorsolateral prefrontal cortex (Figure S3B–D).

### Cognitive similarities between WBS groups

**Figure 2:**
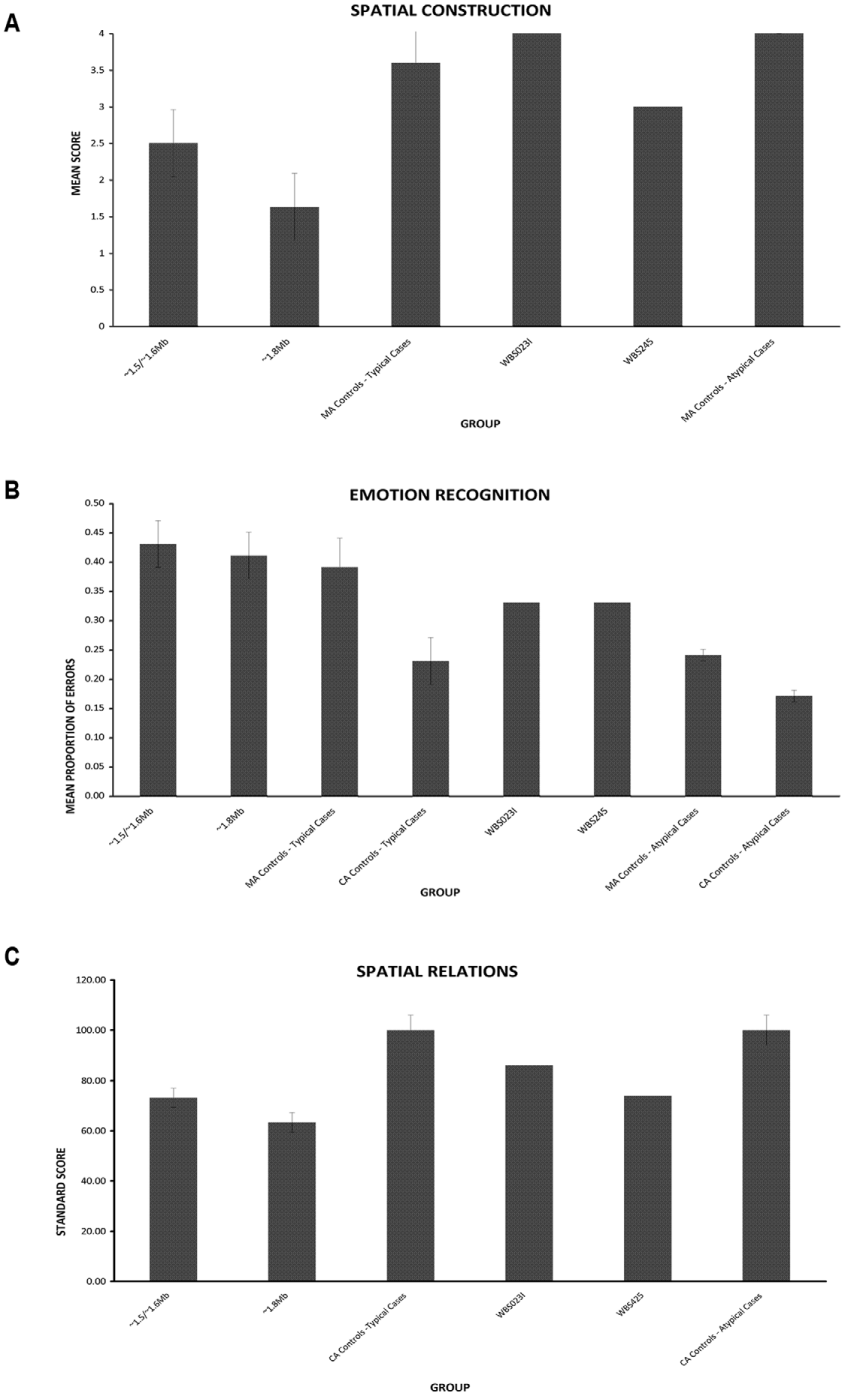
Cognitive similarities and differences between WBS patient cohorts and atypical partial deletion cases. Graphs showing the performance of WBS patients in Groups 1 or 2 (∼1.5/1.6 Mb or 1.8 Mb deletion respectively), patients with atypical deletions, and MA matched and CA matched controls. Differences occur between WBS patient subgroups on spatial perception. Atypical patients show impairments on emotion recognition and spatial perception relative to controls. Error bars represent standard error; * = significant difference at p<.01, ^○^ = marginal difference at p<.05.

**Figure 3:**
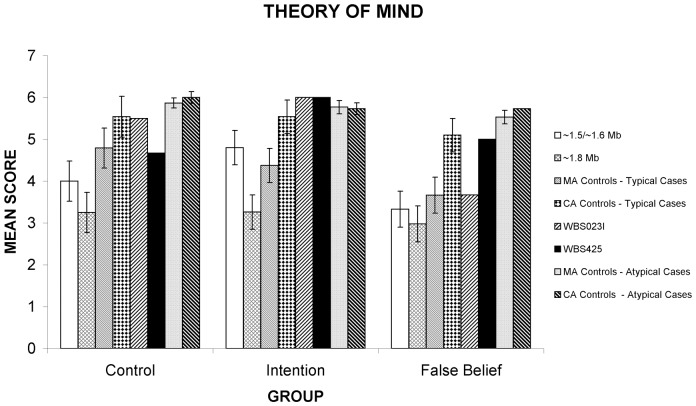
Differences between WBS patient subgroups on understanding of intention. Performance of WBS patients in Groups 1 or 2 (∼1.5/1.6 Mb or 1.8 Mb deletion respectively), patients with atypical deletions, and MA matched and CA matched controls. There are differences between WBS patient subgroups on understanding of Intention and atypical patients show impairments on False Belief relative to controls. Error bars represent standard error; * = significant difference at p<.01, ^○^ = marginal difference at p<.05.

**Figure 4:**
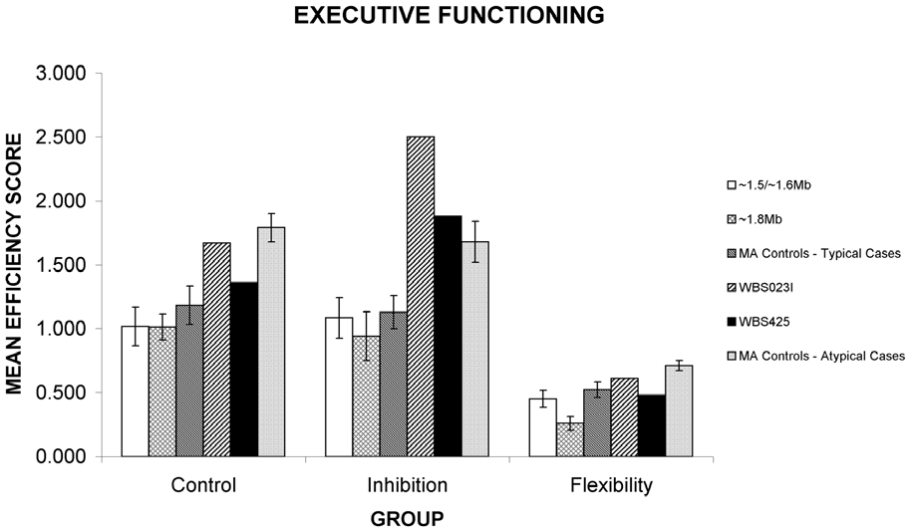
Cognitive similarities and differences between WBS patient cohorts and atypical partial deletion cases. Performance of WBS patients in Groups 1 or 2 (∼1.5/1.6 Mb or 1.8 Mb deletion respectively), patients with atypical deletions, and MA matched and CA matched controls. Differences are seen between WBS patient subgroups on cognitive flexibility. Atypical patients also show impairments on cognitive flexibility relative to controls. Error bars represent standard error; * = significant difference at p<.01, ^○^ = marginal difference at p<.05.

WJ-R COG factor scores were similar for all patient groups (Table 2). Both groups displayed the characteristic WBS cognitive profile, with mild to moderate intellectual impairment, including slowed psychomotor/processing speed, and comparatively good verbal, short-term memory and auditory processing abilities compared to global IQ. All abilities on the WJ-R COG were impaired apart from auditory processing, which usually fell within the borderline impaired to lower average range. Both WBS patient groups displayed a similar performance on measures of spatial construction, emotion recognition, false belief understanding and behavioural inhibition (Figures 2, 3, and 4).

**Table 2:** Descriptive statistics for the WBS patient cohorts and for the atypical partial deletion cases (WBS425 and WBS023I).

	∼1.5/1.6 Mb cohort	∼1.8 Mb cohort	WBS023I	WBS425
Number in group	9	9	1	1
Chronological Age	14.80 (12.00), 5.33–43.67	16.41 (7.72), 6.00–27.25	16.08	23.00
IQ	55 (12), 34–71	50 (15), 32–69	78	69
Mental Age	6.58 (1.39), 4.00–8.67	6.59 (1.52), 4.00–9.33	10.08	9.75
Socio-Economic Status	995 (106), 788–1138	985 (72), 902–1122	1093	917
Gender (F∶M)	5∶4	6∶3	F	F
Hand Dominance (R∶L∶Ambi.)	(6∶3∶0)	(6∶2∶1)	R	Ambi
**WJ-R COG Factor Scores**				
Oral Language	62 (13)	68 (14)	75	77
Long Term Retrieval	69 (12)	74 (11)	80	80
Short Term Memory	70 (11)	68 (14)	98	89
Processing Speed	45 (23)	41 (13)	82	74
Auditory Processing	87 (16)	80 (18)	98	93
Visual Processing	69 (13)	69 (08)	86	61
Comprehension/Knowledge	61 (13)	60 (14)	74	82
Nonverbal Reasoning	67 (14)	66 (10)	71	82

*Note*: Mean (standard deviation) and range are reported for chronological age, IQ, and Socio-Economic Status. Chronological age and mental age are in years. Mean (standard deviation) are provided for WJ-R COG Factor Scores. Standard scores are represented. Standard scores have a mean of 100 and a standard deviation of 15. Scores below 80 are impaired compared to typically developing chronological age matched controls.

**Figure 5:**
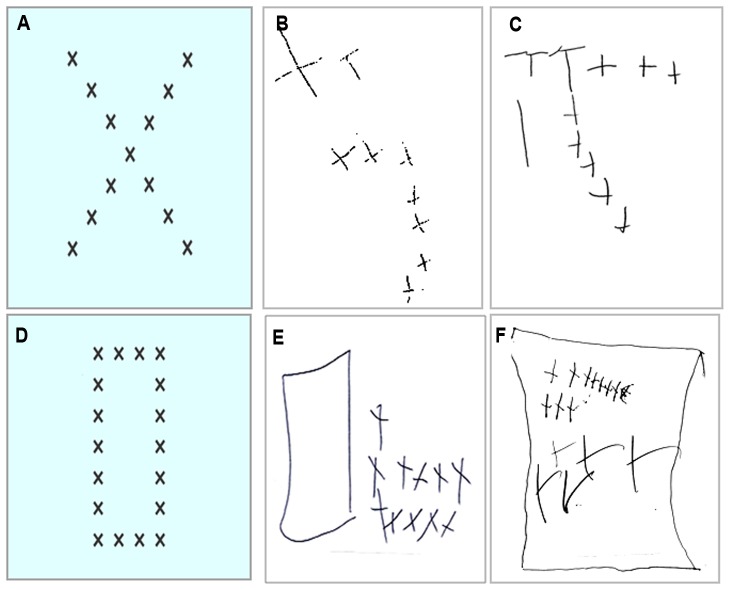
Differences between WBS patient subgroups on spatial construction tasks. Examples of stimuli used in the shape drawing (spatial construction) task (a & d) and example drawings from ∼1.5/1.6 Mb (b & e) and ∼1.8 Mb (c & f) WBS patients. Drawings indicate similar spatial construction deficits in both patient groups, including a local bias (b & c) and spatial integration deficits (b, c, e, & f).

While the initial analysis showed a marginal difference between groups on the spatial construction task *[F (2, 21) = 4.724, p = 0.01]*, follow-up analyses showed that both WBS groups performed similarly to one another *[t(14) = 1.160, p>0.1].* Group 1 patients performed similar to the level of MA-matched controls once the alpha level was adjusted to p<.01 *[t(14) = −1.800, p = .04],* whereas Group 2 performed significantly below MA controls [*t(14) = −3.552, p<0.01]*. Drawings showed qualitatively similar spatial construction deficits across WBS groups, including a local bias and spatial integration impairments (Figure 5).

Analysis of emotion recognition abilities revealed no significant difference across WBS groups and MA-matched controls, but comparison of WBS groups and CA-matched controls was significant *[F (2, 21) = 6.660, p<0.01]*. Both Group 1 *[t(14) = −2.979, p<0.01]* and Group 2 *[t = −3.468, p<0.01]* performed significantly below CA-matched controls on the emotion recognition task (Figure 2B).

For the picture sequencing task, comparison of WBS patients and MA-matched controls on the mechanical (control) aspect showed no difference between the three groups *[F (2, 21) = 2.57, p = 0.05]*. Group 1 performed significantly below CA controls *[t(14) = −2.878 p<0.01]*, as did Group 2 patients *[t(14) = −3.178 p<0.01]*. For false belief stories, WBS patients and MA-matched controls performed similarly in their ability to sequence false belief stories, but both WBS patient groups performed significantly below the level of CA-matched controls *[Group 1, t(14) = −3.527 p<0.01; Group 2, t(14) = −3.511 p<0.01].*

For The Shape School Test, analyses indicated no significant difference across WBS and MA-control groups for the control or the inhibition tasks (Figure 4). As this test was originally designed for preschool children, it was assumed that TD adults would be at ceiling on this task and thus, that WBS patient groups would be well below CA expectations on this task.

### Cognitive differences between WBS groups

Despite similarities in cognitive functioning, there were also cognitive differences between the WBS patient groups (Figures 2C, 3, 4). On the picture sequencing task, there was a significant difference between WBS patients and MA-matched controls on sequencing intention stories *[F (2, 21) = 3.394, p<0.05].* Both Group 1 *[t(14) = −2.202 p = 0.02]* and the MA control *[t(14) = −1.882 p<0.05]* groups marginally outperformed Group 2 in sequencing intention stories (Figure 3), but this was not significant at the adjusted alpha level of p<0.01. There was no difference between Group 1 and MA controls. Similarly, there was a significant difference between the patient groups and CA-matched controls on sequencing intention stories *[F (2, 21) = 7.924, p<0.01]* with the CA control group outperforming Group 2 *[t(14) = −4.112, p<0.01]* but not Group 1.

There was a significant difference across WBS patient groups and MA-matched controls for the flexibility task on The Shape School Test *[F (2, 21) = 5.003 p = 0.01]*. Group 1 marginally outperformed Group 2 *[t(14) = 2.239 p = 0.02]* and controls significantly outperformed Group 2 *[t(14) = −3.202, p<0.01]* (Figure 4). There was no significant difference between Group 1 and MA controls.

On the standardized measure of spatial perception (Test 19 Spatial Relations, WJ-R COG), Group 1 showed marginally greater spatial perceptual skills than Group 2*[t(16) = 2.650, p = 0.01].* All WBS patients performed well below the level of normal controls (control mean±SD = 100±15) (Figure 2C).

### Neuropsychological profile of atypical deletion patients

The neuropsychological profiles of atypical patients WBS023I and WBS425 were explored using the above tests and some additional tests of spatial and executive functioning (Tables 2; Figures 2, 3, and 4; Methods S1). General IQs were slightly higher than the average of both WBS patient groups, but still fell within the classification of an intellectual disability. Both patients displayed relatively high scores on auditory processing and short-term memory, within the low average to average range and consistent with the typical WBS cognitive profile (Table 2). WBS023I displayed relative impairments in oral language, comprehension/knowledge and nonverbal reasoning on the WJ-R COG, and borderline impaired to low average skills on long-term retrieval and processing speed, and WBS425 displayed relative impairments in oral language, processing speed and visual processing and borderline impaired to low average skills on long-term retrieval, comprehension/knowledge and nonverbal reasoning.

Mean performance on Spatial Relations (Test 19) was at least one standard deviation below CA expectations for both patients. Further assessment of spatial abilities suggested impairments in spatial processing relative to TD controls. Both performed in the impaired range on a wide range of spatial measures, including the Benton Judgment of Line Orientation Test [48] (WBS425 <1^st^ percentile, impaired and WBS023I 4^th^ percentile, impaired), the spatial composite score on the DAS [49] (WBS425 <1^st^ percentile, impaired and WBS023I 4^th^ percentile, impaired), and individual DAS subtests - Recall of Designs (WBS425 <1^st^ percentile, impaired and WBS023I 10^th^ percentile, borderline impaired) and Pattern Construction (WBS425 4^th^ percentile, impaired and WBS023I <1^st^ percentile, impaired) (Figure 2C).

WBS023I and WBS425 showed an overall worse performance than MA-matched controls in overall emotion recognition abilities on the DANVA *[t(4) = 2.739, p<0.05]* and displayed difficulties with social reasoning, in particular, understanding of false belief on the picture sequencing task relative to CA controls *[t(4) = −12.513, p<.001 & t(4) = −4.443, p<0.01,* respectively]. WBS425 performed similarly to MA controls but WBS023I performed significantly worse *[t(4) = *−5.181, p<0.01]. In contrast, both patients performed similarly to CA and MA controls in sequencing intention stories. WBS023I performed similarly to CA controls in sequencing mechanical stories, while WBS425 performed more poorly *[t(4) = −4.047, p = 0.1].* WBS023I performed similarly to MA controls in sequencing mechanical stories whereas WBS 425 performed significantly worse *[t(4)* = −3.821, p<0.01] (Figure 3).

In executive functioning abilities, both WBS023I and WBS425 performed worse than the MA-matched control group on the shape school cognitive flexibility subtest *[t(4)* = −2.556, *p<0.05* and *t(4) = *−4.930, *p<0.05,* respectively]. In contrast, they performed similarly to MA controls on the control task and WBS023I performed marginally better *[t(4)* = 2.282, p<0.05] while WBS425 performed similarly to MA controls on the Inhibition task (Figure 4). Further assessment of executive functioning abilities using standardized measures from the DAS, including Matrices (a measure of nonverbal reasoning and Similarities, a measure of verbal abstract reasoning), revealed impairments for both patients. Both performed at the 1^st^ percentile on the Matrices subtest from the DAS and were impaired on the Similarities subtest of the DAS (WBS425, 4^th^ percentile; WBS023I, 2^nd^ percentile). Both performed in the average range on the Face Recognition Test [48], suggesting age-appropriate face perception skills, similar to the typical WBS cognitive profile.

### Impact of deficits on daily functioning

Despite a similar proportion of patients from each group with a reported diagnosis of generalized anxiety or specific phobia (K-SADS-PL), a greater proportion of Group 2 patients were reported with at least one episode of diagnosed depression (75%) compared with Group 1 (25%), but this failed to reach statistical significance. All patients with the larger deletion were described as having obsessions, compared to only half of patients with the typical deletion *[F = 1.732, p<0.05]*.

On the Vineland-II, WBS groups displayed a similar level of adaptive functioning, both overall and across the domains of language, social and daily living skills (Table 3). In contrast, Vineland-II parent interviews indicated significantly more externalizing difficulties *[F (1, 14) = 6.811, p = 0.01]* and marginally more maladaptive behaviours *[F (1, 14) = 3.733, p<0.05]* in Group 2. Group 2 primary caregivers were more likely to endorse items such as “is stubborn”, “is obsessed with objects or activities”, “has strange repetitive habits”, “emotional difficulties”.

**Table 3:** Standard Scores on the Vineland-II Adaptive Behavior Questionnaire.

	∼1.5/1.6 Mb Cohort	∼1.8 Mb Cohort
**Domain**	**Standard Score**	**Standard Score**
Communication	51 (18)	47 (17)
Daily Living Skills	57 (11)	54 (9)
Socialization Domain	59 (13)	58 (9)
Adaptive Behavior Composite	55 (14)	52 (10)
	**V Score**	**V Score**
Internalizing	19 (2)	20 (3)
Externalizing*	15 (2)	17 (2)
Maladaptive Behavior Index○	17 (2)	19 (2)

*Note*: Standard scores have a mean of 100 and s.d. of 15. V scores have a mean of 15 and s.d. of 3.

* = significant difference between groups at p<.01.

○ = marginal difference at p<.05.

## Discussion

To provide a more comprehensive neuropsychological profile in the WBS population and identify additional neurological phenotypes, we sought to compare the genotypic variability of the WBS deletion with the detailed cognitive and behavioural capabilities of patients grouped in the “classical” range based on a positive cytogenetic test. Genetic breakpoint mapping allowed us to divide patients into two subgroups and compare their neuropsychological profiles for detailed genotype-phenotype correlations.

Only two additional genes are deleted in the ∼1.8 Mb cohort (Group 2), *NCF1* and *GTF2IRD2*. The *GTF2I* family of transcription factors are thought to be important for both craniofacial and neurological development [55] and since *GTF2IRD2* has a high degree of structural similarity to GTF2I [56], it may also play a role in cognition and behaviour. Its expression profile supports this prediction, as it is ubiquitously expressed in most body tissues and throughout foetal and adult brain [56]. *NCF1* encodes a component of neutrophil NADPH oxidase, which when mutated causes an immunodeficiency condition with no overt neurological phenotype [57]. Thus, *GTF2IRD2* is the most credible candidate for the neuropsychological differences seen in these patients.

Since both patient subgroups are haploinsufficient for the same core genes we anticipated that all would display the WBS cognitive and behavioural phenotype, but Group 2 would show greater impairments in certain aspects of neuropsychological functions due to loss of an additional transcription factor. Our battery of cognitive tasks was constructed to detect subtle differences concentrating on comparing spatial, social and executive abilities as these are subserved by those regions of the brain known to be abnormal in WBS [14], [15]. We demonstrate that patients whose deletion includes all 3 *GTF2I* family members have additional cognitive impairments involving spatial, social and executive functioning, which may be a consequence of haploinsufficiency for *GTF2IRD2*. Two atypical patients with smaller deletions, but including *GTF2IRD2,* also showed this distinctive neuropsychological profile, providing further evidence of the importance of this gene in this phenotype.

### Brain structural and functional implications

The additional cognitive impairments observed in Group 2 patients may be manifestations of additional neuropathology within the parietal, frontal or cerebellar regions of the brain [9]–[13]. Injury to the dorsolateral prefrontal cortex is associated with deficits in both Theory of Mind and cognitive flexibility [58]–[61], so development of this brain region may be more impaired in larger deletion patients. Indeed, GTF2IRD2 may play a role in the formation of this region during brain development, since *in silico* profiling highlighted expression in the prefrontal cortex in both fetal and adult brains.

### How does impaired executive functioning affect behaviour and psychological functioning?

The general life history reports illustrate that cognitive flexibility impairments have a significant impact upon the daily lives and psychological well-being of patients with WBS, and are greater for those with the larger deletion. The latter were reported with more mood problems such as depression and, in particular, greater externalizing problems, including obsessions and maladaptive behaviours; these psychological characteristics are likely to reflect daily manifestations of executive dysfunction such as poor social reasoning and poor cognitive flexibility. The fact that these difficulties may be a secondary consequence of social or cognitive impairments warrants future research.

### Conclusion

This study identifies cognitive, behavioural and psychological differences between WBS patients with the typical ∼1.5/1.6 Mb size deletion and those with a larger ∼1.8 Mb deletion, and provides the first evidence for a role for GTF2IRD2 in cognition, behaviour and brain development. The GTF2I transcription factors appear to be key to the WBS neurological phenotype but their comp lex and pleiotropic properties means that a clear role for them in human brain development has yet to emerge. Attempts have been made to identify target genes for other family members, namely GTF2IRD1 and GTF2I/TFII-I in the developing rodent brain [62]–[63]. Although no *in vivo* neuronal targets of GTF2IRD1 were detected [62], possible GTF2I targets included genes involved in axon guidance, neurodevelopmental disorders, calcium signaling, cell cycle, and immune response [63]. This supports the hypothesis that these transcription factors are complex proteins, which may be critical regulators of other transcription factors, histone deacetylases, and signaling molecules. GTF2IRD2 may also have a putative role in some of these pathways, however, target genes that are specific to GTF2IRD2 may be the ones that cause executive dysfunction.

Of course the mechanism(s) involved in producing these neurological phenotypes may be more complex than just haploinsufficiency. The majority of the literature discounts parent-of-origin effects in WBS [64], but there are reports of parent-of-origin effects on microcephaly and growth [65], as well as on *GTF2I* expression [66], both of which may be related to partial imprinting. More evidence and research is required into epigenetic control mechanisms in WBS, and future studies should consider not only which genes are deleted, but which parent the intact chromosome is from and what variants are present in these genes.

Findings from this study and others with partial deletion patients [21]–[25] suggest an additive effect in deleting all three *GTF2I* family genes, leading to more severe neurological phenotypes. Our findings that all WBS patients demonstrated impairments in spatial, executive and social functioning support brain imaging and post-mortem studies which demonstrate abnormalities in the areas of the brain that subserve these functions. Further degrees of deficit in cognitive flexibility, social reasoning and spatial manipulation in ∼1.8 Mb deletion patients are consistent with prefrontal, parietal and perhaps cerebellar deficits, and it is possible that the extent of neurological abnormality may be greater in these patients. Twin studies suggest that executive functioning skills are highly heritable psychological traits [67], and we show that *GTF2IRD2* may be one of those genes that contribute to executive function. This paves the way for further investigations into the biological underpinnings of executive functions and the mechanisms involved.

Our findings also show the importance of genetics-led investigations into screening for more subtle underlying phenotypes. We show that cognitive, behavioural, psychological and most likely neurological phenotypes vary amongst patients with ‘classical’ WBS, depending on the size of their genetic deletion and propose that detailed genotyping of patients is essential prior to testing to avoid heterogeneous results. Such studies will advance our understanding of the nature and extent of the neurological phenotypes associated with WBS as well as the role of the genes involved. They may also aid the development of therapies to enhance the management of WBS neuropsychological deficits, reduce their functional impact and aid independence and emotional wellbeing.

## Supporting Information

Methods S1 **Supplementary Methods.**
(DOC)

Figure S1 **Example item from Test 19, Spatial Relations on the WJ-R COG.** This task requires the participant to select which component parts are needed to make up a particular shape. The shapes are initially geometrical, but become more abstract as item difficulty increases. This task measures spatial skills, but importantly, unlike the Pattern Construction Task, does not involve a psycho-motor or constructional component.
(DOC)

Figure S2 **Example a) Control/Mechanical and b) Intention stories from Langdon et al. (1997)'s nonverbal picture sequencing task, measuring Theory of Mind abilities.** Note, in order to sequence the latter story appropriately, one must understand something about the mother's beliefs and intentions. That is, she realizes it is her son's birthday and she goes to the shop with the intent of buying her son a birthday present.
(DOC)

Figure S3 **In silico analysis of GTF2IRD2 Gene Expression in Human Brain.**
(DOC)

Table S1 **Descriptive Statistics for Normal Controls.**
(DOCX)

Table S2 **Genetic profile of the 55 WBS patients.**
(DOC)
