# Supplementary material for: A Role for Transcription Factor GTF2IRD2 in Executive Function in Williams-Beuren Syndrome
Source: PLoS One. 2012 Oct 31;7(10):e47457. doi: 10.1371/journal.pone.0047457 (PMC3485271; doi:10.1371/journal.pone.0047457)
Supplement: Methods S1 — Supplementary Methods. (DOC) [file pone.0047457.s001.doc]

**Porter et al 2012**

**Supplementary Information**

**METHODOLOGY**

**Genetic Analyses**

*Real-time quantitative PCR.*

Real-time quantitative polymerase chain reaction (PCR) assays were performed to map the deletion breakpoints in the WBS patients and to confirm array-CGH data. A panel of 60 targeted probes were designed to span the whole WBSCR and extend into the LCR flanking regions (primer sequences available on request). Primers were designed using Beacon Designer software (Premier Biosoft) and optimized to allow multiplex amplification at 55/60^o^C. SYBR Green JumpStart Taq ReadyMix (Stratagene, UK) was used to quantify gene copy number in patient DNA samples. Primers within the *CFTR* gene were used to correct for any differences in DNA concentration between samples. PCR amplification efficiencies were previously determined for each primer pair from the slope of a standard curve using a serial dilution of control DNA, where PCR efficiency=10(-1/slope)-1. PCR reactions were carried out in a MX3000P qPCR machine (Stratagene) in 96-well optical plates with a final reaction volume of 10ul. 5 ng of genomic DNA and gene-specific primers at a final concentration of 200 nM were used with cycling conditions of: 95°C denaturation for 10 minutes followed by 40 cycles at 95°C for 30 seconds, 60°C 1 minute and 72°C 1 minute. This was followed by 1 minute at 95°C, 30 seconds at 55/60°C and a final step of 30 seconds at 95°C to obtain a dissociation curve. Reactions were carried out in triplicate, and an inert dye (ROX) was used to correct any differences in reaction volume between wells. Results were analysed with MX3000P comparative quantification software, and expressed as relative values, compared to a control DNA sample. The starting copy number of the unknown sample was determined using the comparative Ct method, as previously described [1].

*Array-Comparative Genome Hybridisation Microarray analysis*

Array Comparative Genome Hybridisation (aCGH) analysis was performed using commercial oligonucleotide microarrays. Protocols were as per manufacturer’s instructions. Agilent high-resolution custom Human genome 244K arrays were used, containing 236,381 distinct features and an average resolution of about 10 kb (Human Genome CGH Microarray. Agilent Technologies, Santa Clara, California). Also CytoSure Syndrome ISCA v2 arrays (OGT) comprising 60,000 probes, which target 498 genome regions and have a resolution of one probe every 60kb were used. Agilent DNA Analytics software with optimized algorithms was used to analyse the aCGH data. Structural variations are genomic alterations that involve segments of DNA >1kb. All copy number changes detected were screened against the Database of Genomic Variants (http://projects.tcag.ca/variation/) and ENSEMBL (http://www.ensembl.org/index.html) databases to determine their CNV status. Those CNVs with pathological implications were validated using locus-specific real-time QPCR in the patient and both parents to determine their mode of inheritance.

**Neuropsychological Measures**

1. *General Intellectual and Specific Cognitive Abilities: Woodcock-Johnson Test of Cognitive Ability – Revised*

#### Materials

Participants were administered the Woodcock-Johnson Tests of Cognitive Ability – Revised or WJ-R COG [2] to assess general intellectual and more specific areas of cognitive functioning. Although the Differential Ability Scales or the British equivalent - the British Ability Scales - has been the most popular measure of intellectual and spatial ability for WBS patients in the past, we chose to use the WJ- R COG, so that the same test battery could be used for children and adults or for patients with extremely low or extremely high cognitive ability, allowing for direct comparisons between these patients. The WJ-R COG is a widely used test battery with respectable psychometric properties and normative data for persons aged 2 to 95 years of age, making the battery appropriate for all participants in the current study on the basis of both chronological age and mental age (IQ) and, therefore, minimizing the chance of floor effects. Development of the WJ-R COG was theoretically driven; the battery is based on the Horn-Cattell Gf-Gc theory, proposing two types of intelligence called fluid (Gf) and crystallized (Gc) intelligence, or innate and learned intelligence [3].

There are 21 tests in the WJ-R COG, 7 core and 14 supplemental. A combination of test scores from these 21 tests is used to derive scores for eight cognitive domains, these include: 1) *Oral Language -* A global measure of verbal capabilities including both language expression and language comprehension; 2) *Long Term Retrieval -* Measures the ability to spontaneously recall auditory-visual associations (verbal labels for pictorial stimuli) following a delay; 3) *Short Term Memory -* Measures immediate memory span for auditory verbal information (phonological short-term memory); 4) *Processing Speed -* Measures speed of information processing and psychomotor speed, but also captures attention, impulse control and visual scanning; 5) *Auditory Processing -* Measures the ability to comprehend patterns among auditory stimuli, such as changes in length, sequencing and pitch; 6) *Visual Processing -* Measures visual perception, recognizing degraded visual stimuli, and immediate memory span for nonverbal (visual) information; 7) *Comprehension/Knowledge* - Measures breadth and depth of general (or cultural-specific) knowledge, often referred to as “Crystallized Intelligence”, also captures verbal capabilities and verbal reasoning, 8) *Nonverbal Reasoning -* Measures the ability to reason in novel situations (where culture-specific facts are irrelevant) , often referred to as “Fluid or Innate Intelligence” . For further details, see the WJ-R COG Examiner’s Manual [4]. In addition, performance on the 7 core tests derives a general ability score, similar to ‘full-scale IQ’ on the Wechsler tests and the DAS.

Administration and Scoring

Tests were administered according to standardized instructions provided in the WJ-R COG Examiner’s Manual [4]. The battery was administered over two separate sessions, no longer than one week apart. On average, the battery took three to four hours in total to administer (two hours per session with breaks).

Data was scored manually using the procedure outlined in the WJ-R COG Examiner’s Manual [4] and was then checked using the computerized WJ-R COG scoring system.

1. *Spatial Construction*

Materials

Congruent and incongruent shape designs (such as those outlined in 5, and displayed in Figure S2) were displayed on a computer screen as bitmap files (approximately 12cm X 12 cm in size and centered on the screen).

Administration and Scoring

Designs were displayed on the screen for as long as the participant required and participants were asked to copy the designs as carefully and as closely as possible. Participants copied 4 designs (2 congruent and 2 incongruent).

Each drawing was scored by a research assistant who was blind to the purpose of the study. Scoring was as follows: Drawings scored 0 if the small and large shape were drawn separately on the page and 1 if the small and large shape were correctly integrated. This provided a minimum score of 0 and a maximum score of 4 for each participant (because there were 4 drawings in total). An independent judge scored the drawings and inter-rater agreement was 96%.

1. *Emotion Recognition: The Diagnostic Analysis of NonVerbal Accuracy Scale*

Materials

We used the Diagnostic Analysis of NonVerbal Accuracy (DANVA) scale [6] to measure emotion recognition. Data from numerous studies with over 2000 children has established very good reliability and validity of this measure, even for children as young as 3 years of age (the manual is constantly updated and is available at <http://www.psychology.emory.edu/clinical/interpersonal/DANVA/manual03.doc> ). This is a psychometrically sound, well standardized and widely used test battery including 24 adult faces, 24 child faces and 32 adult postures in the form of standard size colour photographs, as well as tapes containing 24 adult and 24 child voices saying “I’m going out of the room now, but I’ll be back later” in various emotional expressions (happy, sad, angry and scared). For the posture images, the entire body is shown, but the face is colored black so it cannot be used to determine the emotional expression. The posture images include an additional eight neutral expressions, which were of no interest in the present study. There are an equal number of happy, sad, angry and scared expressions in each modality (face, voice and posture).

Administration and Scoring

The DANVA task was administered according to the standardized instructions [6]. In brief, participants were shown expressions in the following order: adult faces, child faces, adult voices, child voices and then gestures; they were asked to say whether each expression was happy, sad, angry or scared (forced choice). Stimuli within a modality were also presented in a fixed order; emotional expressions were randomised within each mode of expression. Error rates were recorded for each expression in each modality (adult faces, child faces, adult paralanguage, child paralanguage, postures).

There were no significant interactions containing the variable Group, so the mean proportion of errors was averaged across Modality and Emotion for each participant and a total emotion recognition score was used in comparative statistical analyses.

1. *Social Reasoning: The Nonverbal Picture Sequencing Task*

Materials

We chose not to use Happè’s classic verbal Theory of Mind (ToM) tasks, because findings in the literature using these measures with WBS patients remains contradictory, and because we have found that WBS patients often tend to use their strong verbal skills to pass these tests, despite having a ToM impairment. Instead, we chose to use a nonverbal picture sequencing task to measure ToM. This task has been used previously with WBS patients, and was sensitive to ToM deficits in some WBS patients, regardless of their verbal or intellectual levels of ability [7].

The picture sequencing task used in the present study was designed by Langdon et al. [8] to test ability to reason about psychological causality in people with schizophrenia. The task was based on an earlier Baron-Cohen, Leslie and Frith study used to assess ToM in children with autism, Down syndrome and normal preschool children [9]. There are mental state stories, as well as social script and mechanical stories. In addition to the critical false-belief stories, stories were also designed to assess understanding of pretence, unrealized goals and intention (see below). The idea is that if a person has a selective ToM deficit, they should experience difficulty sequencing some or all of the mental state stories, but not the social script or mechanical stories.

Twenty-four stories were depicted in 4-card picture sequences (21cm x 15cm) using a simple black and white cartoon style. Example stimuli are shown in Figure S3. There were eight story types, with three examples per story type:

1. Mechanical 1: Objects interacting causally with each other.
2. Mechanical 2: A person and objects interacting causally.
3. Social script 1: A single person acting out an everyday social routine.
4. Social script 2: More than one person interacting in everyday social routines.
5. Pretence: One or more persons involved in pretend play.
6. False-belief: A person, unaware of an event in a story, acts on a false belief.
7. Unrealized-goal: Goal directed activity, where the goal is not achieved.
8. Intention: Goal-directed activity, where initial actions could be associated with multiple goals.

Administration and Scoring

The picture sequencing task was administered in accordance with Langdon et al. [8]. After two practice items, stories were presented in a random order. Cards for each story (four in total) were placed in a random order presented face down. Participants were instructed to turn the cards over and to put them in order so they told a story ‘that made sense’. There was no time limit. Order of cards was recorded for each story.

The picture sequencing task was scored using Langdon et al.’s criterion[8]. In short, each sequence scored two points if the first card was positioned correctly, two points if the last card was positioned correctly, and one point each for correct second and third card placement. Scores ranged form zero to six. A score of zero was also provided when a participant failed to produce a sequence. Scores were averaged across the three examples of a story type (range 0-6).

There were no significant interactions containing the variable Group, so in accordance with Langdon et al. [8], stories were collapsed into four story categories:

1. Mechanical (Mean of Mechanical 1 and Mechanical 2 Stories);

2. Social (Mean of Social Script 1 and Social Script 2);

3. False belief (Mean of Pretence and False belief stories); and

4. Intention (Mean of Unrealized-goal and Intention stories).

1. *Executive Functioning: The Shape School Test*

Materials

Executive abilities were assessed using the Shape School Test, a measure of both response inhibition and flexibility [10].

Administration and Scoring

The Shape School test was administered in accordance with procedures outlined in Espy [10]. In brief, participants were shown three rows of five colored shapes and circles, depicted as pupils in ‘The Shape School’. First the participants were asked to name the pupils’ color (Control Condition). Second the participants were asked to inhibit salient, but irrelevant information and name the colors only for those shapes with a happy facial expression; they were told to ignore the sad pupils (Inhibition Condition). Third the participants were required to switch between naming colors and naming shapes, depending on whether the shape was wearing a hat (Shift Condition). In the final condition (Both Inhibition and Shifting), the participants were required to name the colors if pupils were not wearing a hat and to name the shapes if pupils were wearing a hat (shift), but to only name those pupils with a happy facial expression (inhibition).

Importantly, in line with Espy [10], pretests were conducted to ensure that participants could accurately name all colors (red, blue and yellow), both shapes (circle and square) and both expressions (happy and sad), and to ensure that participants were able to accurately distinguish between those pupils who were and who were not wearing a hat.

Accuracy and naming speed were recorded for each condition and efficiency scores were calculated as outlined in Espy [10]. In brief, efficiency scores were calculated using the following formula [efficiency = (the number correct – the number of errors) / total time].

#### *6. Vineland-II Adaptive Behavior Scales – Second Edition*

#### Materials

The Vineland Adaptive Behavior Scales – 2^nd^ Edition (Parent Survey) [11] interview was conducted with a parent of each of the WBS patients to assess qualitative and quantitative differences between the two WBS patient groups in their day-to-day functioning. The Vineland Adaptive Behavior Scales – 2^nd^ Edition is used extensively, both clinically and in research. It has respectable psychometric properties and normative data for persons aged 0 to 90 years of age, again making the measure appropriate for all participants in the current study on the basis of both chronological age and mental age (IQ) and, therefore, minimizing the chance of floor effects.

There are 3 main adaptive functioning domains: 1) *Communication –* assessing receptive, expressive and written language skills; 2) *Daily living Skills -* Measures personal and domestic skills as well as community engagement; 3) *Socialization -* Measures interpersonal relationships, play and leisure activities and coping skills. In addition, average performance across these domains derives an Adaptive Behavior Composite score. Two behavior domains are also included: *1) Internalizing -* and 2) *Externalizing -* , as well as a global Maladaptive Behavior Index which is a composite of internalizing, externalizing and additional maladaptive behavior items. For further details, see the Vineland Adaptive Behavior Scales – 2^nd^ Edition (Parent Survey) Manual [11].

Administration and Scoring

The interview was administered according to standardized instructions provided in the Examiner’s Manual [11]. The administrator was a clinician in training who was blind to the purpose and subgroups in the study.

Data was scored using the procedure outlined in the Examiner’s Manual [11] and was then checked using the computerized scoring system.

#### *7. Schedule for Affective Disorders and Schizophrenia (K-SADS)*

The Schedule for Affective Disorders and Schizophrenia for School-Age Children-Present and Lifetime Version (K-SADS-PL; 12] was administered to primary caregivers to assess lifetime history of psychopathology according to DSM-IV criteria.

The K-SADS-PL has been used extensively for diagnosing Axis I disorders in typically developing children [13; 14] and has been used successfully with intellectually impaired populations [15; 16] and people with WBS [17]. The K-SADS-PL also has excellent psychometric properties, including an interrater reliability that is highly competitive with other diagnostic interviews, and has been validated against a number of other popular measures [18]. The interviewer was a research assistant, blind to the purpose of this study and to the WBS subgroups.

Administration and Scoring

The interviewer completed appropriate training provided by the authors of the K-SADS-PL. The interview was administered and scored according to standardized training procedures (K-SADS-PL; 12).

#### Supplementary Materials – Partial Deletion Cases

1. *Benton Facial Recognition Test*

The Benton Facial Recognition test was used to assess face recognition abilities in our atypical partial deletion patients [19]. Participants are shown a target face and must find that identity amongst an array of distracters below. We used the long version, with 22 items.

This test is commonly used for clinical and research purposes and has been used extensively in the WBS literature where people with WBS consistently seem to display a relative strength on this task [20].

Administration and Scoring

The test was administered and scored using information provided in the test manual [19].

1. *Benton Judgment of Line Orientation Test*

The Benton Judgment of Line Orientation test was used to assess spatial perception in our atypical partial deletion patients [21]. Participants are shown target lines pointing in a particular direction at a specific orientation and must match these target lines to those with exactly the same orientation; these targets are placed next to similar distracters. We used the long version, with 30 items in total. This test is commonly used for clinical and research purposes and has been used within the WBS literature where people with WBS consistently perform poorly on this task, or at least no better than their mental age [22, 23].

Administration and Scoring

The test was administered and scored using information provided in the test manual [21].

1. *Differential Ability Scales*

The Differential Ability Scales (or DAS) is a popular measure of intellectual ability with very respectable psychometric properties [24]. The DAS played an integral role in the identification and development of the Williams-Beuren Syndrome cognitive profile [25].

The DAS provides a Global Ability Score, comparable to a Full Scale IQ, as well as three composite scores: 1) *Verbal –* assessing vocabulary and verbal abstract reasoning; 2) *Nonverbal Reasoning -* Measures nonverbal abstraction and sequential and quantitative reasoning; 3) *Spatial -* Measures spatial construction skills and visual memory. Four subtests of particular interest for the current study were: 1) *Recall of Designs –* participants are exposed to visual designs and must reproduce them from memory using pencil and paper; 2) *Pattern Construction –* Participants rotate blocks to match spatial designs; 3) *Matrices-* Participants choose the design missing from an abstract pattern and 4) *Similarities-* Participants must describe how three things are alike, with items starting very concrete and becoming quite abstract.

Administration and Scoring

The test was administered and scored according to guidelines outlined in the examiner’s manual [24].

**Supplementary References**

1. Livak KJ (1997) Comparative Ct method. ABI Prism 7700 Sequence Detection System. User Bulletin no. 2. PE Applied Biosystems.

2. Woodcock RW, Johnson MB (Eds.) (1989) Woodcock-Johnson psycho-educational battery - revised: Itasca, IL: Riverside Publishing.

3. Horn JL, Noll J (1997) Human cognitive abilities: Gf-Gc theory. In: Flanagan DP, Genshaft JL, Harrison PL, editors. Contemporary intellectual assessment: Theories, tests, and issues. New York: Guilford p. 53-91.

4. Woodcock, RW, Mather N (1989) WJ-R tests of cognitive ability - standard and supplemental batteries: Examiner's manual, Woodcock-Johnson Psycho-Educational Battery - Revised: Itasca, IL: Riverside Publishing.

5. Porter MA, Coltheart M. (2006) Global and local processing in Williams, autistic and Down syndrome: Perception, attention and construction. Dev Neuropsychol 30: 771-789.

6. Nowicki S, Duke MP (1994) Individual differences in the nonverbal communication of affect: the diagnostic analysis of nonverbal accuracy scale. J Nonverbal Behav 18: 9-35.

7. Porter MA, Dodd H, Cairns D (2008) Psychopathological and behavior impairments in Williams-Beuren syndrome: The influence of gender, chronological age and cognition. Child Neuropsychol 15: 359-374.

8. Langdon R, Michie PT, Ward PB, McConaghy N, Catts SV, et al. (1997) Defective self and/or other mentalising in Schizophrenia: A cognitive neuropsychological approach. Cogn Neuropsychiatry 2: 167-193.

9. Baron-Cohen S, Leslie AM, Frith U (1985) Does the autistic child have a theory of mind? Cognition 21: 37-46.

10. Espy KA (1997) The shape school: Assessing executive function in preschool children. Dev Neuropsychol 13: 495–499.

11. Sparrow S, Cicchetti D, Balla D (2005) Vineland Adaptive Behavior Scales (2nd ed.). Minneapolis: Pearson Assessment.

12. Kaufman J, Birmaher B, Brent D, Rao U, Flynn C, et al. (1997) Schedule for affective disorders and schizophrenia for school-age children-present and lifetime version (k-sads-pl): Initial reliability and validity data. J Am Acad Child Adolesc Psychiatry 36: 980-988.

13. Cortes AM, Saltzman KM, Weems CF, Regnault HP, Reiss AL, et al. (2005). Development of anxiety disorders in a traumatized pediatric population: A preliminary longitudinal evaluation. Child Abuse Neglect 29: 905–914.

14. Hakko H, Lintunen J, Lappalainen J, Makikyro T, Rasanen P, et al. (2006). Nicotine use and dependence and their association to psychiatric disorders in a large sample of adolescent psychiatric inpatients. Addict Behav 31: 1873–1880.

15. Antshel KM, Fremont W, Roizen NJ, Shprintzen R, Higgins AM, et al. (2006) ADHD, Major Depressive Disorder, and Simple Phobias are prevalent psychiatric conditions in youth with Velocardiofacial syndrome. J Am Acad Child Adolesc Psychiatry 45: 596–603.

16. Masi G, Brovedani P, Mucci M, Favilla L (2002) Assessment of anxiety and depression in adolescents with mental retardation. Child Psychiat Hum D 32: 227–237.

17. Dodd H and Porter M (2008) Psychopathology in Williams syndrome: The effect of individual differences across the life span. J. Men. Health Res. Int. Dis. 2, 89-109.

18. Kaufman J, Schweder AE (2004) The schedule for affective disorders and schizophrenia for school-age children: Present and lifetime version (K-SADS-PL). In: Hilsenroth MJ, Segal DL, editors. Comprehensive handbook of psychological assessment. Hoboken, NJ: Wiley; p. 247-255.

19. Benton AL, Hamsher K, Varney NR, Spreen O (1983a) Benton test of facial recognition. New York: Oxford University Press; 1983a.

20. Bellugi U, Lichtenberger L, Jones W, Lai Z, St. George M (2000) The neurocognitive profile of Williams syndrome: A complex pattern of strengths and weaknesses. J
Cognitive Neurosci 12: 7–29.

21. Benton AL, Hamsher K, Varney NR, Spreen O (1983b) Benton judgment of line orientation test. New York: Oxford University Press.

22. Bellugi U, Sabo H, Vaid J (1988) Spatial deficits in children with Williams syndrome. In: Stiles-Davis J, Kritchevsky M, Bellugi U, editors. Spatial cognition: Brain bases and development. Hillsdale, NJ: Lawrence Erlbaum Associates p. 273-298.

23. Wang PP, Doherty S, Rourke SB, Bellugi U (1995) Unique profile of visuo-perceptual skills in a genetic syndrome. Brain Cognition 29: 54-65.

24. Elliot CD (1990) Differential Ability Scales. New York: The Psychological Corporation.

25. Mervis CB, Robinson BF, Bertrand J, Morris CA, Klein-Tasman BP, et al. (2000) Armstrong SC. The Williams syndrome cognitive profile. Brain Cognition 44: 604–628.
