# Supplementary material for: A Role for Transcription Factor GTF2IRD2 in Executive Function in Williams-Beuren Syndrome
Source: PLoS One. 2012 Oct 31;7(10):e47457. doi: 10.1371/journal.pone.0047457 (PMC3485271; doi:10.1371/journal.pone.0047457)
Supplement: Figure S1 — Example item from Test 19, Spatial Relations on the WJ-R COG. This task requires the participant to select which component parts are needed to make up a particular shape. The shapes are initially geometrical, but become more abstract as item difficulty increases. This task measures spatial skills, but importantly, unlike the Pattern Construction Task, does not involve a psycho-motor or constructional component. (DOC) [file pone.0047457.s002.doc]

***Figure S1:*** Example item from Test 19, Spatial Relations on the WJ-R COG. This task requires the participant to select which component parts are needed to make up a particular shape. The shapes are initially geometrical, but become more abstract as item difficulty increases. This task measures spatial skills, but importantly, unlike the Pattern Construction Task, does not involve a psycho-motor or constructional component.
