# Supplementary material for: A Role for Transcription Factor GTF2IRD2 in Executive Function in Williams-Beuren Syndrome
Source: PLoS One. 2012 Oct 31;7(10):e47457. doi: 10.1371/journal.pone.0047457 (PMC3485271; doi:10.1371/journal.pone.0047457)

***Supp. Figure 2:*** Example a) Control/Mechanical and b) Intention stories from Langdon et al. (1997)’s nonverbal picture sequencing task, measuring Theory of Mind abilities. Note, in order to sequence the latter story appropriately, one must understand something about the mother’s beliefs and intentions. That is, she realizes it is her son’s birthday and she goes to the shop with the intent of buying her son a birthday present.

a)


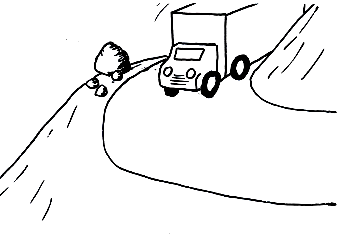

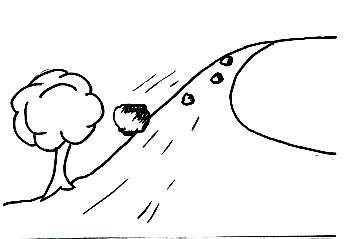

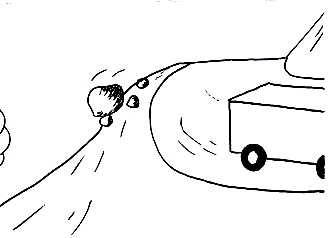

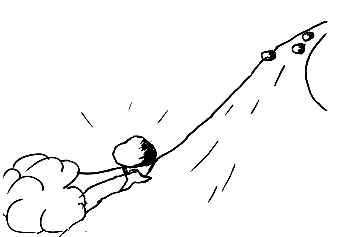


b)


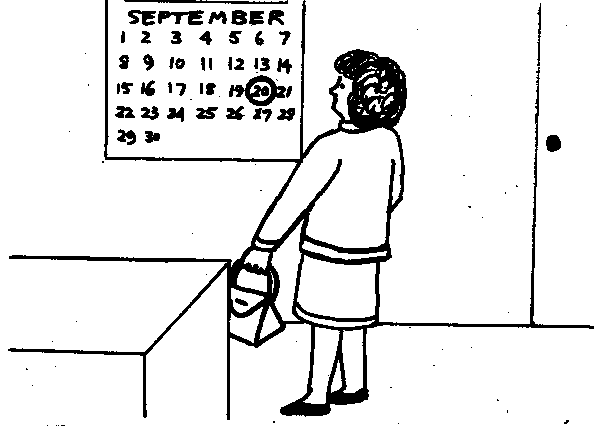

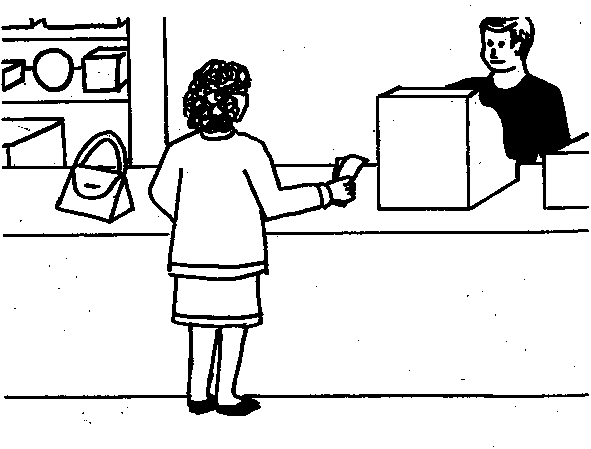

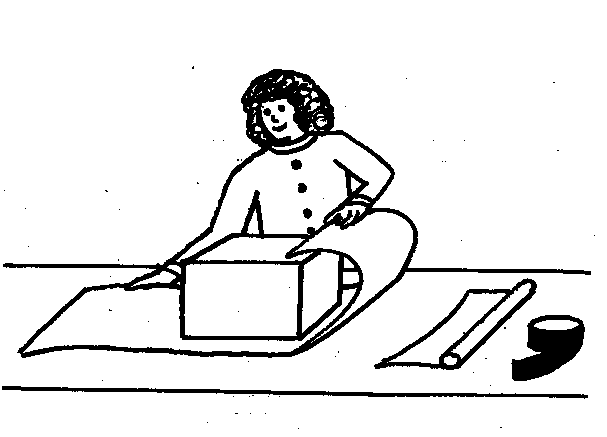

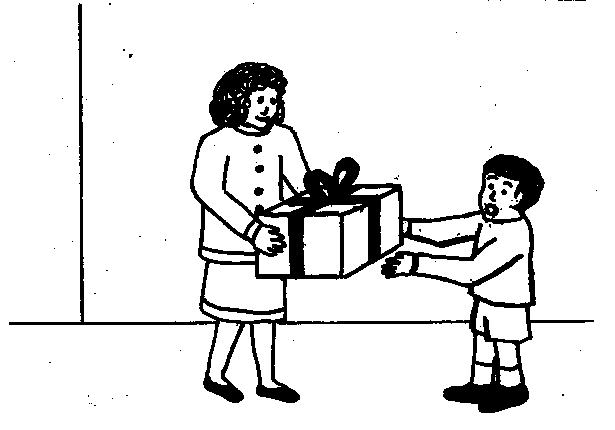

Supplement: Figure S2 — Example a) Control/Mechanical and b) Intention stories from Langdon et al. (1997)'s nonverbal picture sequencing task, measuring Theory of Mind abilities. Note, in order to sequence the latter story appropriately, one must understand something about the mother's beliefs and intentions. That is, she realizes it is her son's birthday and she goes to the shop with the intent of buying her son a birthday present. (DOC) [file pone.0047457.s003.doc]
