# Supplementary material for: A Role for Transcription Factor GTF2IRD2 in Executive Function in Williams-Beuren Syndrome
Source: PLoS One. 2012 Oct 31;7(10):e47457. doi: 10.1371/journal.pone.0047457 (PMC3485271; doi:10.1371/journal.pone.0047457)
Supplement: Figure S3 — In silico analysis of GTF2IRD2 Gene Expression in Human Brain. (DOC) [file pone.0047457.s004.doc]

***Figure S3. In silico analysis of GTF2IRD2 Gene Expression in Human Brain***

1. Expression of GTF2IRD2 in normal tissues from 3 normal adults; data reanalysed from from GEO - GDS3113/214852

***
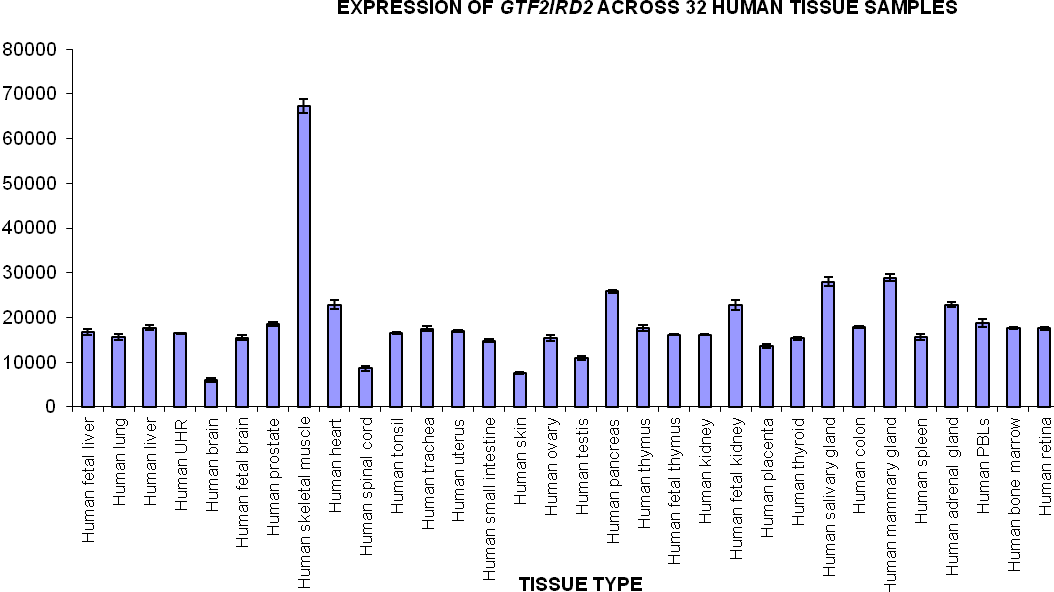
***

1. Data reanalysed from: GEO- GDS2190/215569_

***
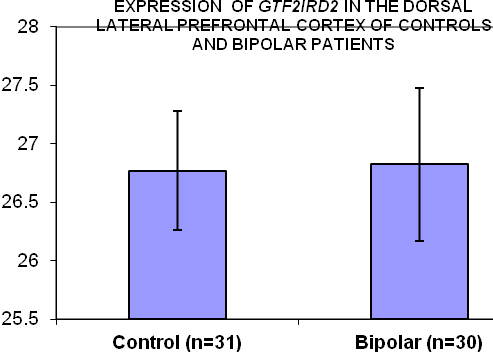
***

1. Data reanalysed from: GDS2191/215569_

***
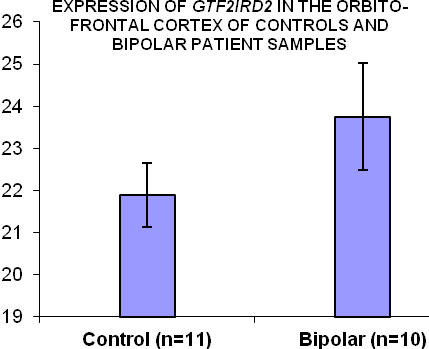
***

1. Data reanalysed from: GDS1917/228765_

***
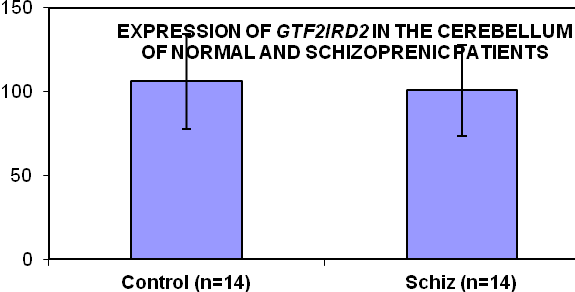
***
