# Supplementary material for: A Role for Transcription Factor GTF2IRD2 in Executive Function in Williams-Beuren Syndrome
Source: PLoS One. 2012 Oct 31;7(10):e47457. doi: 10.1371/journal.pone.0047457 (PMC3485271; doi:10.1371/journal.pone.0047457)
Supplement: Table S1 — Descriptive Statistics for Normal Controls. (DOCX) [file pone.0047457.s005.docx]

**Table S1**

**Descriptive Statistics for Normal Controls**

|  | Typically Developing Controls | Comparison btw WBS and Control Groups |
| --- | --- | --- |
|  | Chronological Age |  |
| Shape School Test (n=8) | 5.65 (1.06) 3.92 – 8.67 | F (2,21) = .268, p > .1 |
| Picture Sequencing Task (n = 8) | 5.96 (0.64) 4.66 – 6.67 | F (2,21) = .685, p > .1 |
| Spatial Construction (n = 8) | 6.10 (1.46) 4.00 – 8.67 | F (2,21) = .268, p > .1 |
| DANVA (MA Controls) (n = 8) | 5.86 (1.48) 4.00 – 8.17 | F (2,21) = .202, p > .1 |
| DANVA (CA Controls) (n = 8) | 15.72 (9.81) 5.58 – 32.00 | F (2,21) = .011, p > .1 |

**Note:** Mean (S.D.), range; CA = Chronological Age; MA = Mental Age. On these measures there were only 8 individuals from each WBS patient group who participated. For the ~1.5/1.6 Mb WBS group, mean CA was 19.25, standard deviation 12.75, range 5.33 – 43.67 and mean MA was 5.97, standard deviation 1.49, range 4.00 – 8.67. For the ~1.8Mb WBS group, mean CA was 16.81, standard deviation 8.52, range 6.00 – 27.25 and mean MA was 6.23, standard deviation 1.53, range 4.00 – 9.33.
