# Supplementary material for: A Role for Transcription Factor GTF2IRD2 in Executive Function in Williams-Beuren Syndrome
Source: PLoS One. 2012 Oct 31;7(10):e47457. doi: 10.1371/journal.pone.0047457 (PMC3485271; doi:10.1371/journal.pone.0047457)
Supplement: Table S2 — Genetic profile of the 55 WBS patients. (DOC) [file pone.0047457.s006.doc]

**Table S2**

**Genetic profile of the 55 WBS patients**

| **Patient ID** |  | **FISH** | **Size of deletion#** | **WBSCR** | **distal gene** |
| --- | --- | --- | --- | --- | --- |
|  |  | **test** | **~ Mb** | **deletetion** | **deleted** |
| 184KKK |  | +ve | 1.5 | Bc-Bm | *GTF2I* |
| 097GG |  | +ve | 1.5 | Bc-Bm | *GTF2I* |
| 002A |  | +ve | 1.62 | Bc-Bm | *GTF2I* |
| 008C |  | +ve | 1.62 | Bc-Bm | *GTF2I* |
| 009D |  | +ve | 1.62 | Bc-Bm | *GTF2I* |
| 059T |  | +ve | 1.62 | Bc-Bm | *GTF2I* |
| 078Z |  | +ve | 1.62 | Bc-Bm | *GTF2I* |
| 084BB |  | +ve | 1.62 | Bc-Bm | *GTF2I* |
| 103II |  | +ve | 1.62 | Bc-Bm | *GTF2I* |
| 106JJ |  | +ve | 1.62 | Bc-Bm | *GTF2I* |
| 109KK |  | +ve | 1.62 | Bc-Bm | *GTF2I* |
| 130RR |  | +ve | 1.62 | Bc-Bm | *GTF2I* |
| 133SS |  | +ve | 1.62 | Bc-Bm | *GTF2I* |
| 142VV |  | +ve | 1.62 | Bc-Bm | *GTF2I* |
| 157BBB |  | +ve | 1.62 | Bc-Bm | *GTF2I* |
| 160CCC |  | +ve | 1.62 | Bc-Bm | *GTF2I* |
| 163DDD |  | +ve | 1.62 | Bc-Bm | *GTF2I* |
| 166EEE |  | +ve | 1.62 | Bc-Bm | *GTF2I* |
| 190MMM |  | +ve | 1.62 | Bc-Bm | *GTF2I* |
| 196OOO |  | +ve | 1.62 | Bc-Bm | *GTF2I* |
| 037M |  | +ve | 1.64 | Bc-Bm | *NCF1* |
| 040N |  | +ve | 1.64 | Bc-Bm | *NCF1* |
| 091EE |  | +ve | 1.64 | Bc-Bm | *NCF1* |
| 148XX |  | +ve | 1.64 | Bc-Bm | *NCF1* |
| 175HHH |  | +ve | 1.64 | Bc-Bm | *NCF1* |
| 115MM |  | +ve | 1.64 | Bc-Bm | *NCF1* |
| 118NN |  | +ve | 1.64 | Bc-Bm | *NCF1* |
| 121OO |  | +ve | 1.64 | Bc-Bm | *NCF1* |
| 124PP |  | +ve | 1.64 | Bc-Bm | *NCF1* |
| 127QQ |  | +ve | 1.64 | Bc-Bm | *NCF1* |
| 136TT |  | +ve | 1.64 | Bc-Bm | *NCF1* |
| 139UU |  | +ve | 1.64 | Bc-Bm | *NCF1* |
| 075Y |  | +ve | 1.64 | Bc-Bm | *NCF1* |
| 004B | ** | +ve | 1.5 | Bc-Bm | *GTF2I* |
| 030J | ** | +ve | 1.54 | Bc-Bm | *NCF1* |
| 072X | ** | +ve | 1.54 | Bc-Bm | *NCF1* |
| 081AA | ** | +ve | 1.54 | Bc-Bm | *NCF1* |
| 022H | ** | +ve | 1.62 | Bc-Bm | *GTF2I* |
| 046P | ** | +ve | 1.62 | Bc-Bm | *GTF2I* |
| 051Q | ** | +ve | 1.62 | Bc-Bm | *GTF2I* |
| 066V | ** | +ve | 1.62 | NOL1R-Bm | *GTF2I* |
| 019G | ** | +ve | 1.64 | Bc-Bm | *NCF1* |
| 178III | ** | +ve | 1.64 | Bc-Bm | *NCF1* |
| 012E | ** | +ve | 1.8 | Bc-Bm | *GTF2IRD2* |
| 032K | ** | +ve | 1.8 | Bc-Bm | *GTF2IRD2* |
| 043O | ** | +ve | 1.8 | Bc-Bm | *GTF2IRD2* |
| 054R | ** | +ve | 1.8 | Bc-Bm | *GTF2IRD2* |
| 056S | ** | +ve | 1.8 | Bc-Bm | *GTF2IRD2* |
| 062U | ** | +ve | 1.8 | Bc-Bm | *GTF2IRD2* |
| 069W | ** | +ve | 1.8 | Bc-Bm | *GTF2IRD2* |
| 196OOOb | ** | +ve | 1.8 | Bc-Bm | *GTF2IRD2* |
| 112LL | ** | +ve | ~2 | Bc-Bt | *GTF2IRD2B* |
| 187LLL | ** | +ve | ~3.3 | *CALN1*-Bt | *GTF2IRD2B* |
| WBS023I | ** | -ve | ~0.78 | LIMK1-Bm | *GTF2IRD2* |
| WBS425 | ** | -ve | ~0.78 | LIMK1-Bm | *GTF2IRD2* |
